# Challenges in the retrospective assessment of trauma: comparing a checklist approach to a single item trauma experience screening question

**DOI:** 10.1186/s12888-016-0720-1

**Published:** 2016-02-01

**Authors:** Souci Frissa, Stephani L Hatch, Nicola T Fear, Sarah Dorrington, Laura Goodwin, Matthew Hotopf

**Affiliations:** Academic Department of Psychological Medicine, King’s College London, Institute of Psychiatry, Psychology & Neuroscience, BRC nucleus, Mapother House, De Crespigny Park, London, SE5 8AF UK; Academic Department of Military Mental Health and King’s Centre for Military Health Research, King’s College London, London, UK

**Keywords:** Trauma, Appraisal of trauma, Stressful life events, Post traumatic stress disorder

## Abstract

**Background:**

Research on trauma and its impact on mental health typically relies on self-reports which can be influenced by recall bias and an individual’s subjective interpretation of events. This study aims to compare responses on a checklist of life events with a trauma experience screening question, both of which assessed trauma experience retrospectively.

**Methods:**

A community sample of adults were asked about life events from a checklist before asking them whether they ever had a trauma experience, i.e. “an event that either puts them or someone close to them at risk of serious harm or death”.

**Results:**

Less than half of the sample who reported at least one life event on the checklist that qualified as a trauma reported a trauma experience that they perceived put them or close others at risk of serious harm. Women responders, those reporting early life traumas, and a greater number of lifetime trauma events were more likely to report a trauma experience. Current symptoms of Common Mental Disorder did not account for differences in reporting of trauma experiences.

**Conclusions:**

Epidemiological approaches which require participants to make subjective judgement on the severity of the trauma experience will capture individual differences that we have shown are influenced by gender and previous trauma experience.

## Background

Compared to people without a trauma history, trauma survivors are at higher risk of many problems including depression, substance abuse, suicidal behaviour, physical illnesses and poverty [1–3]. One of the possible consequences of trauma is posttraumatic stress disorder (PTSD), an often severe and disabling condition with a lifetime prevalence in the general population of up to 9 % [4–11]. Unlike other mental illnesses, the diagnosis of PTSD requires not only symptoms but also a cause; that is, the occurrence of a trauma event which posed an actual or threat of death, serious injury or sexual violence in one of the following ways: directly experiencing, witnessing, learning that it occurred to close family member/friend or repeated exposure to aversive details of trauma events [12]. Epidemiological studies of PTSD typically rely on a subjective assessment by the participant and/or researcher as to whether a particular event was sufficiently severe to justify being a “trauma”. However, in practice this may be difficult to determine. Recall bias [13, 14], an individual’s subjective interpretation of events [15] and type of the event itself [8] may strongly influence whether an individual who has experienced an event sees it as traumatic.

Although exposure to trauma events is widespread, affecting 21–89 % of the population in their lifetime [7–11, 16], only a minority will go on to develop a mental disorder. Experiencing an unpleasant event as a trauma is not an objective process. The trauma experience is filtered through cognitive processes relating to the appraisal of an event which may be perceived by some, but not all, individuals as a severe threat [15]. Cognitive appraisal is also an important part of the stress response mechanism [17]. Theoretical models have posited the role of cognitive appraisals as mediators in the development and persistence of trauma-related psychopathology [18]. Among survivors of traumas, negative appraisals of the meaning of the trauma appear to account for symptoms of depression and posttraumatic stress beyond objective measures like frequency and severity of trauma [19].

One of the factors influencing retrospective accounts of life events is current mental health status. Cohort studies of people who were exposed to the same traumatic event have reported that the retrospective report of the trauma was affected by current mental health. Severity of PTSD symptoms at follow-up are associated with an increase in retrospective accounts of the frequency and severity of exposure to trauma events. Those doing better, with a lower severity of symptoms at follow-up reported the event as less disturbing than they previously had [20–22]. A similar association was also observed with worsening self-rated perception of health and endorsement of new trauma experience at follow-up [23].

Research into factors influencing the recollection of trauma events has so far mainly focused on highly selective samples, such as individuals who suffered childhood sexual abuse [24–26] or combat-related trauma [20, 22]. Most of these studies assessed the consistency of reports of traumatic events on repeated assessments over a period of time. However, to the best of our knowledge, the consistency of reports obtained using different methods to assess trauma experience has never been done in a community sample.

In the present study, participants were asked about trauma experiences twice within the same interview. First, we used a checklist of life events and participants were asked whether they had experienced each of the events on the checklist. With this approach all events were treated as equivalent. Second, participants were asked as part of a PTSD checklist whether they had ever experienced an event that either put them or someone close to them at risk of serious harm or death (i.e. a “trauma” in the meaning of the PTSD questionnaire). This approach allowed respondents to appraise the experience of the trauma event and give a subjective judgement to the experience [27]. The aims of the present study are to assess how these two different responses correspond, and to examine which factors are associated with reporting a trauma experience in those who previously reported a specific event on the life event checklist. We hypothesise that individuals with a current common mental disorder will be more likely to report a trauma experience than those without.

## Methods

### Study design and participants

The data came from the South East London Community Health (SELCoH) study, a cross-sectional survey of 1698 adults from randomly selected households in the south London boroughs of Southwark and Lambeth. Trained interviewers conducted face-to-face interviews in the participant’s home [28]. Professional translators, booked through the South London and Maudsley (SLaM) National Health Service trust, were used in interviews with non-English speaking adults. The sample was comparable to the UK Census information for the same area with regards to demographic and socioeconomic indicators. A detailed description of the methods is available elsewhere [28].

### Measures

#### Socio-demographic indicators

The socio-demographic indicators used in the analysis include gender, ethnic group, age, relationship status and migration status. Self-reported ethnicity indicated identification with one of the following groups: White, Black African, Black Caribbean, Asian or Other. Migration status was captured as born in the UK or not. The socioeconomic indicators in the analysis were: employment status, household income and educational attainment. Employment status was classified into the following six categories: employed (full time or part time), student, unemployed, sick or disabled, retired and looking after the home with children. Participants reported annual household income before deductions for income tax and National Insurance based on the following five categories; (1) £0–£5475, (2) £5476–£12,097, (3) £12,098–£20,753, (4) £20,754–£31,494 and (5) £31,495 or more. Educational attainment was classified into the following groups; no qualifications, up to GCSE level or equivalent, up to Advanced level or equivalent (high school equivalent), Higher (university) degree or above.

#### Life events

Life events were measured by self-reported experiences of 11 listed stressful events on a checklist. The events were selected using a combination of different checklist measurements from the literature on stressful experiences relevant to inner city populations [29, 30].

For this study, we selected the six lifetime events captured on a checklist that qualified as traumatic events according to Diagnostic and Statistical Manual of Mental Disorders (DSM-5) [12].

Participants were asked whether or not they experienced the following events in their lifetime: witnessed violence to someone (Have you ever seen something violent happen to someone (e.g., attacked or beaten) or seen someone killed?); experienced a serious accident; lived in a combat or war zone or a political uprising; victim of a serious crime (Have you ever been attacked, mugged, robbed or been the victim of a serious crime?); injured with a weapon (Has anyone ever injured you with a weapon – gun, knife, stick, etc.?); and physical or sexual abuse. The number of different events were summed to create a cumulative life event variable.

To account for the type of life event, we categorised events into two groups. The first group included directly experienced interpersonal violence: victim of a serious crime; injured with a weapon; and physical or sexual abuse. The second group included all other events: witnessed violence to someone; experienced a serious accident; lived in a combat or war zone or a political uprising. These categories do not indicate the severity of the trauma or their impact on a person’s mental health.

#### Early life events

Early life events were events before the age of 16 years measured by self-reported experiences of 9 listed events on a checklist [29, 30]. For this study, we selected the three life events before the age of 16 years that qualified as a trauma event according to DSM-5. Participants were asked on a checklist whether or not the following events occurred before the age of 16 years: major illness or accident that required hospital admission for a week or more; physical abuse that left a bruise or mark; and sexual abuse. Responses were then categorised into a binary variable, of those who had and had not reported an early life events.

#### Trauma experiences

Following the life event checklist, a lifetime DSM-IV Criterion A1 event screening question was used as a screening to the Primary Care PTSD screen (PC-PTSD) questionnaire [31]. Participants were asked whether they had ever experienced at any time in their lifetime ‘an event that put them or close others at risk of serious harm or death’ [32]. Responses were categorised as a binary variable.

Those who reported a lifetime trauma experience were then asked the questions from the PC-PTSD questionnaire which is a 4-item measure developed by National Centre for PTSD [31]. The PTSD symptoms were not analysed in this study as they would only have been completed if individuals had reported a lifetime trauma experience.

#### Common mental disorder

Common Mental Disorder (CMD) was assessed by the Revised Clinical Interview Schedule (CIS-R), which is a structured interview that asks about 14 symptom domains (fatigue, sleep problems, irritability, worry, depression, depressive ideas, anxiety, obsessions, subjective memory and concentration, somatic symptoms, compulsions, phobias, physical health worries and panic). A total CIS-R score of 12 or more was used to indicate the overall presence of CMD [33].

### Analytic strategy

The total sample (*N* = 1698) was analysed to calculate weighted prevalences of the specific items from the life event checklist and for the trauma experience screening item. The weighted prevalence for the overlap between these measures was then calculated.

Logistic regression analyses examined the associations between reporting events as a trauma experience with the socio-demographic indicators, characteristics of life events and current CMD. These analyses were restricted to participants who reported at least one life event checklist item (*N* = 1241). We reported the odds ratios (OR) with 95 % confidence intervals (CI), with trauma experience as the dependent variable. Models adjusted for gender and age (as a continuous variable) are presented for all logistic regression models.

The independence of the associations between the socio-demographic indicators, life events, CMD and the odds of reporting a trauma experience were tested through successive addition of factors in a multivariable logistic regression model. These analyses were restricted to participants who reported at least one life event checklist item (*N* = 1241). In model I, we only included the socio-demographic indicators. In model II, in addition to the items in model I we adjusted for life event variables (experience of life event before the age of 16 years, cumulative life events and type of life event). In model III, to test the hypothesis that current CMD is associated with the odds of reporting a trauma experience, we further included CMD status in addition to the items in model II.

All statistical analyses were undertaken using the statistical software package STATA (version 11) [34]. We used survey commands (svy) for estimates of prevalence and associations. This analysis accounted for clustering by household, and data were weighted for non-response bias within households. Frequencies were not weighted. Odds ratios (OR) with 95 % confidence intervals were calculated to determine prevalence estimates and associations.

### Ethical approval

The study received approval from the King’s College London research ethics committee, reference CREC/07/08-152.

A written informed consent for participation in the study was obtained from participants.

## Result

### The lifetime prevalence of exposure to life events and the proportion of participants who reported a trauma experience (*N* = 1698)

A total of 1241 participants (72.1 %) reported at least one life event that met the criteria for trauma according to DSM-5. More than 40 % of the participants (41.5 % (95 % CI 39.0–44.0)) had witnessed violence; more than a third (37.7 % (95 % CI 35.1–40.3)) had been a victim of serious crime; a quarter (28.5 % (95 % CI 26.2–30.8)) of the sample reported lifetime physical or sexual abuse; a fifth (21.9 % (95 % CI 19.8–24.0)) had experienced a serious accident; approximately a tenth (13.9 % (95 % CI 12.0–15.8)) had lived in combat or war zone and a tenth (10.8 % (95 % CI 9.2–12.3)) reported injury with a weapon.

Out of the total population of 1681 who completed both the life events checklist and a lifetime trauma experience screening question; more than a third (39.3 % (95 % CI 36.8–41.8)) reported a specific item from life event checklist but did not report a trauma experience; in contrast to 33.4 % (95 % CI 31.0–35.9) who reported both a specific item from life event checklist and a trauma experience screening item. A fifth of the population (23.5 % (95 % CI 21.3–25.7)) did not report any of the items from life events checklist and did not report a trauma experience screening item; and a small proportion (3.8 %(95 % CI 2.8–4.8)) of participants did not report a life event checklist item but did report a traumatic experience screening item (the result tables showing the total sample are available on request from the authors).

### Factors associated with reporting a trauma experience among those who had experienced an event from the life event checklist

#### Comparison by socio-demographic indicators

There was a significant association between age and reporting a trauma experience, with higher odds in the 45–54 and 55–64 year age groups compared to the 16–24 year age group. The odds of reporting a trauma experience was higher in the never married and previously married group compared to the married or cohabiting. Those who were unemployed and participants who were not working because of health reasons were more likely to report trauma experience compared to the employed group. However, the retired group were less likely to report a trauma experience than the employed group. Participants from the lowest household income group were more likely to report a trauma experience compared to the high income group. These associations remained significant after adjustment for age and gender (Table 1).

**Table 1:** The prevalence estimates and the associations for reporting a trauma experience by socio-demographic indicators in individuals who reported a life event on the checklist (*N* = 1241)

Socio-demographic characteristics		Reported a traumatic experience		
	N (%)	Prevalence (95 % CI)	OR (95 % CI)	AOR^a^ (95 % CI)
Gender				
Female	656 (52.9)	47.6 (43.6–51.6)	1.2 (1.0–1.5)	1.2 (1.0–1.5)
Male	585 (47.1)	43.1 (39.0–47.3)	1	1
Ethnic group				
White	781 (63.0)	47.3 (43.4–51.1)	1	1
Black-Caribbean	110 (8.9)	46.8 (37.0–56.7)	1.0 (0.6–1.5)	1.0 (0.6–1.5)
Black-African	164 (13.2)	40.2 (32.5–47.9)	0.8 (0.5–1.1)	0.7 (0.5–1.0)
Asian	39 (3.2)	32.6 (18.1–47.0)	0.5 (0.3–1.1)	0.5 (0.3–1.0)
Other	145 (11.7)	48.0 (39.4–56.6)	1.0 (0.7–1.5)	1.0 (0.7–1.4)
Age (years)				
16–24	260 (21.0)	41.5 (35.3–47.6)	1	1
25–34	288 (23.2)	45.2 (39.2–51.2)	1.2 (0.8–1.6)	1.2 (0.8–1.6)
35–44	246 (19.8)	48.0 (41.5–54.4)	1.3 (0.9–1.9)	1.3 (0.9–1.8)
45–54	204 (16.4)	52.8 (45.6–59.9)	1.6 (1.1–2.3)*	1.5 (1.1–2.3)*
55–64	116 (9.4)	58.7 (49.5–68.0)	2.0 (1.3–3.2)**	2.0 (1.3–3.2)**
65+	127 (10.2)	32.7 (23.8–41.6)	0.7 (0.4–1.1)	0.7 (0.4–1.1)
Relationship status				
Never married	504 (40.6)	48.8 (44.2–53.4)	1.3 (1.0–1.7)*	1.3 (0.9–1.7)
Married/cohabiting	551 (44.4)	41.6 (37.1–46.2)	1	1
Divorced/separated/widowed	186 (15.0)	50.7 (43.2–58.3)	1.4 (1.0–2.1)*	1.5 (1.0–2.1)*
Migration status				
Non migrant	762 (63.5)	46.2 (42.3–50.1)	1	1
Migrant	439 (36.5)	45.7 (40.8–50.7)	1.0 (0.8–1.3)	1.0 (0.8–1.2)
Employment status				
Employed	666 (53.9)	45.5 (41.4–49.6)	1	1
Student	173 (14.0)	42.2 (34.5–49.9)	0.9 (0.6–1.2)	0.9 (0.6–1.4)
Unemployed	134 (10.9)	55.8 (46.8–64.9)	1.5 (1.0–2.3)*	1.5 (1.0–2.3)*
Sick and disabled	65 (5.3)	69.9 (58.1–81.6)	2.8 (1.6–5.0)**	2.7 (1.5–4.8)**
Retired	136 (11.0)	35.4 (26.9–44.0)	0.7 (0.4–1.0)*	0.6 (0.3–1.0)
Looking after children	61 (4.9)	42.5 (30.0–54.9)	0.9 (0.5–1.5)	0.8 (0.5–1.5)
Yearly household income				
£0–£5475	108 (10.2)	60.2 (50.5–69.9)	1.8 (1.1–2.8)*	1.8 (1.1–2.8)*
£5476–£12,097	160 (15.2)	45.7 (37.3–54.0)	1.0 (0.7–1.4)	1.0 (0.7–1.5)
£12,098–£20,753	144 (13.6)	46.7 (37.8–55.5)	1.0 (0.7–1.5)	1.0 (0.7–1.5)
£20,754–£31,494	132 (12.5)	41.3 (32.6–50.0)	0.8 (0.5–1.2)	0.8 (0.5–1.2)
£31,495 or more	512 (48.5)	46.2 (41.6–50.8)	1	1
Educational attainment				
No qualifications	170 (13.8)	44.4 (36.1–52.8)	0.9 (0.6–1.3)	0.9 (0.6–1.4)
Up to GCSE level	249 (20.3)	43.8 (37.4–50.2)	0.9 (0.6–1.2)	0.9 (0.6–1.2)
Advanced level	315 (25.7)	47.1 (41.4–52.7)	1.0 (0.8–1.4)	1.0 (0.7–1.3)
Higher degree or above	494 (40.2)	46.7 (42.1–51.4)	1	1

Frequencies are unweighted and may not add up due to missing values

*OR* odds ratio, *AOR* adjusted odds ratio

^a^Adjusted for age (continuous) and gender

*p < 0.05

**p < 0.01

#### Comparison by life event characteristics

Compared to the group who did not report a life event before the age of 16 years, the odds of reporting a lifetime trauma experience were doubled for those who did. There was also a strong, graded relationship between the cumulative number of life events and the odds of reporting a traumatic experience (test for trend *P* < 0.001). Participants who experienced events categorised as interpersonal violence had increased odds of reporting a traumatic experience. These associations remained significant in the models adjusted for age and gender (Table 2).

**Table 2:** The prevalence estimates and the associations for reporting a trauma experience by life event variables and current CMD in individuals who had reported a life event on the checklist (*N* = 1241)

Stressful life events		Reported a trauma experience		
	N (%)	Prevalence (95 % CI)	OR (95 % CI)	AOR^a^ (95 % CI)
Early life event (before 16 years old)				
No	659 (53.1)	36.6 (32.8–40.5)	1	1
Yes	582 (46.9)	56.7 (52.4–61.0)	2.3 (1.8–2.9)***	2.3 (1.8–2.9)***
Cumulative number of life events				
1 event	459 (37.1)	31.1 (26.5–35.7)	1	1
2 events	350 (28.3)	39.7 (34.3–45.1)	1.5 (1.1–2.0)*	1.6 (1.1–2.1)**
3 events	250 (20.2)	62.4 (56.0–68.8)	3.7 (2.6–5.2)***	4.1 (2.9–5.9)***
4 events	124 (10.0)	75.8 (68.2–83.4)	6.9 (4.4–11.0)***	8.1 (5.1–13.0)***
5 or more events	55 (4.4)	85.2 (75.7–94.6)	12.7 (5.8–27.8)***	15.0 (6.6–34.0)***
Type of life event				
Directly experienced interpersonal violence	888 (71.6)	50.7 (47.1–54.3)	2.0 (1.5–2.6)***	2.0 (1.5–2.6)***
Other events	353 (28.4)	34.5 (29.2–39.7)	1	1
Common Mental Disorder (CIS–R ≥12)				
No	911 (73.7)	41.1 (37.6–44.5)	1	1
Yes	326 (26.4)	59.1 (53.4–64.7)	2.1 (1.6–2.7)***	2.0 (1.5–2.7)***

Frequencies are unweighted and may not add up due to missing values

*OR* odds ratio, *AOR* adjusted odds ratio

^a^Adjusted for age (continuous) and gender

*p < 0.05

**p < 0.01

***p < 0.001

#### Comparison by current common mental disorder status

Participants who met the criteria for CMD at the time of the interview had increased odds of reporting a traumatic experience in both the unadjusted analyses and models adjusted for age and gender (Table 2).

### Multivariable associations between reporting a trauma experience, socio-demographic indicators, stressful life events and CMD

Female gender, never being married, experiencing life event before the age of 16 years and the cumulative number of life events were strongly associated with the odds of reporting a traumatic experience, independent of other socio-demographic indicators, the type of life event and current symptoms of CMD. The association between type of trauma and reporting a trauma experience attenuated when adjusted for life event before the age of 16 years and cumulative life events.

There was no association between current CMD and reporting a traumatic experience in the models adjusted for socio-demographic and life event indicators (Table 3).

**Table 3:** Odds ratios (OR) for reporting a traumatic experience in fully adjusted models

	Model I	Model II	Model III
Gender			
Female	1.1 (0.8–1.4)	1.5 (1.1–2.0)**	1.5 (1.1–2.0)*
Male	1	1	1
Ethnicity			
White	1	1	1
Black-Caribbean	1.0 (0.6–1.6)	1.1 (0.7–1.9)	1.2 (0.7–2.0)
Black-African	0.8 (0.5–1.2)	0.8 (0.5–1.3)	0.8 (0.5–1.4)
Asian	0.8 (0.4–1.6)	0.9 (0.4–2.0)	0.9 (0.4–2.0)
Other	1.0 (0.6–1.6)	1.0 (0.6–1.6)	1.0 (0.6–1.7)
Age			
16–24	1	1	1
25–34	1.0 (0.6–1.7)	1.0 (0.6–1.7)	1.0 (0.6–1.7)
35–44	1.3 (0.8–2.2)	1.2 (0.7–2.2)	1.3 (0.7–2.3)
45–54	1.8 (1.1–3.2)*	1.7 (0.9–3.0)	1.7 (0.9–3.1)
55–64	1.7 (0.9–3.3)	1.5 (0.8–3.0)	1.6 (0.8–3.1)
65+	0.8 (0.3–2.1)	0.8 (0.3–2.1)	0.9 (0.3–2.2)
Marital status			
Never married	1.6 (1.1–2.2)**	1.5 (1.0–2.2)*	1.5 (1.0–2.2)*
Married/cohabiting	1	1	1
Divorced/separated/widowed	1.2 (0.8–1.9)	1.2 (0.8–1.9)	1.2 (0.8–1.9)
Migration status			
Non migrant	1	1	1
Migrant	1.0 (0.7–1.4)	1.0 (0.8–1.4)	1.0 (0.8–1.4)
Employment status			
Employed	1	1	1
Student	1.0 (0.6–1.7)	0.9 (0.5–1.6)	0.9 (0.5–1.7)
Unemployed	1.4 (0.9–2.4)	1.3 (0.8–2.3)	1.3 (0.7–2.2)
Sick and disabled	1.9 (0.9–3.9)	1.7 (0.8–3.6)	1.6 (0.8–3.3)
Retired	1.0 (0.5–2.1)	0.9 (0.4–1.9)	0.9 (0.4–1.9)
Looking after children	1.1 (0.6–2.1)	1.3 (0.7–2.5)	1.3 (0.7–2.4)
House hold income			
£0–£5475	1.5 (0.8–2.6)	1.3 (0.7–2.3)	1.2 (0.7–2.2)
£5476–£12,097	1.0 (0.6–1.7)	1.0 (0.6–1.7)	1.0 (0.6–1.7)
£12,098–£20,753	1.1 (0.7–1.7)	1.1 (0.7–1.7)	1.0 (0.6–1.6)
£20,754–£31,494	0.8 (0.5–1.2)	0.8 (0.5–1.2)	0.7 (0.4–1.2)
£31,495 or more	1	1	
Educational status			
No qualifications	0.8 (0.4–1.4)	0.7 (0.4–1.3)	0.8 (0.4–1.4)
Up to GCSE level	0.7 (0.5–1.1)	0.7 (0.4–1.1)	0.7 (0.4–1.1)
Advanced level	1.0 (0.7–1.4)	0.8 (0.6–1.2)	0.9 (0.6–1.2)
Higher degree or above	1	1	1
Early life event (before 16 years old)			
No		1	1
Yes		1.6 (1.2–2.1)**	1.6 (1.2–2.1)**
Cumulative stressful life events			
1 event		1	1
2 events		1.5 (0.9–2.2)	1.4 (0.9–2.1)
3 events		3.6 (2.3–5.6)***	3.5 (2.2–5.4)***
4 events		5.7 (3.2–10.3)***	5.6 (3.1–10.0)***
5 or more events		11.8 (4.7–29.6)***	11.6 (4.6–29.2)***
Type of Life event			
Directly experienced interpersonal violence		0.8 (0.6–1.2)	0.8 (0.6–1.2)
Other events		1	1
Common Mental Disorder			
No			1
Yes			1.2 (0.9–1.7)

Model I contains all socio–demographic indicators

Model II contains life event variables in addition to variables in model I

Model III contains CMD in addition to variables in model II

*p < 0.05

**p < 0.01

***p < 0.001

## Discussion

Exposure to trauma in South East London is considerably higher than in other studies of inner city populations in Europe (21 % in Munich and 28 % in Zurich) [7, 9]. In this study the majority (72.1 %) of the sample reported at least one item from the checklist of life events that qualified as trauma [12]. However, less than half (46.0 %) of those who reported a trauma item on the checklist reported a trauma experience they perceived put them or close others at risk of serious harm. Unlike the checklist which allows participants to endorse events without a rating of severity or the threat that the event posed, participants are required to exercise their judgement of the severity of a threat on the trauma experience screening question.

Women were more likely to report a trauma experience. This could be due to a gender difference in post trauma cognitions (meaning-making) which was suggested by previous studies as a possible explanation for gender differences in PTSD [19]. It could also be due to a gender difference in the type of trauma experienced, with women experiencing more interpersonal trauma than men [19]. However, a post hoc analysis revealed that the number reporting interpersonal trauma was similar between women (70.9 % (95 % CI 67.2–74.5) and men (71.2 % (95 % CI 67.4–75.0)) and there was no gender difference in reporting trauma experience by type of life event (the results are available on request from the authors). The higher likelihood for women to endorse traumatic experiences could partly explain the observed higher prevalence of PTSD among women reported in several studies, including the previous report from the same population whereby men were more likely to report a trauma event, but women were more likely to experience symptoms of PTSD [11]. This supports the argument that the high prevalence of PTSD in women may not be due to an increased overall risk of trauma, but due to a greater vulnerability to the effects of trauma events [4, 8, 16, 35, 36].

We did not observe the hypothesised difference in reporting a trauma experience between those with current common mental disorder and those without (after adjustment). The fact that the association between the experience of life event before the age of 16 years, cumulative life event and reporting a trauma experience did not diminish after accounting for mental disorder, suggests that they are directly linked and not mediated by current mental health state. Although several follow up studies have reported an association between current mental health state and how people remember their traumatic experience, these studies did not consider the effects of cumulative and childhood trauma which are more likely in individuals with mental disorder [20–22].

We observed a strong association between cumulative life events captured on a checklist and reporting a trauma experience. The negative appraisal of an event may not only depend on the experience of an individual event, but also on previous exposure to trauma [18]. Greater trauma exposure relates to more negative cognitions about oneself and the world which predicts how individuals interpret and make sense of their experience [19]. This may provide one explanation for the association observed in previous studies between previous exposure to trauma event and posttraumatic stress disorder (PTSD) resulting from subsequent trauma [35, 36].

Our findings highlight an association between exposure to stressful life events during childhood and reporting a trauma experience. This could be due to the severity of the events included in the childhood stressful life events questions, or that victims of child abuse are more likely to experience trauma as an adult [37], or alternatively that they are more likely to perceive subsequent stressful events as a traumatic experience [18]. Reporting stressful events before the age of 16 years may also be a marker for an individual’s early life environment, e.g. neglect, which may also play a role in emotional development and later responses to trauma. This is consistent with findings from other studies in the general population sample and in combat veterans that reported those who experienced childhood trauma had a higher risk of PTSD in adulthood from subsequent trauma [5, 35, 38].

In the PTSD literature a wide range of events such as spousal infidelity [6], marital disruption [39], miscarriage [40] and childbirth [41] have also been reported to be associated with the onset of PTSD even though they may not all meet the DSM criteria for a trauma. In the current study we also found a small proportion (4 %) of participants who reported a trauma experience even though they did not report any of the events from the life event checklist which met this criterion. Whilst this may be partly explained by the limitation of the life event checklist which is not exhaustive, it is possible that they had experienced other life events which would not meet the criteria for trauma. On the other hand, many individuals do not develop PTSD despite the experience of extreme life threatening trauma like torture under captivity as prisoner of war [42]. Some studies have reported the importance of subjective measures of perceived threat as a better predictor of PTSD symptoms than objective measures of danger, including in studies of torture survivors [43], burn victims [44], and accident survivors [45]. These studies showed that the appraisal of the threat was a key determinant of whether an event triggers PTSD or not and the stress response also depends on whether the stressor is appraised as threatening or not [17]. This could be translated to other mental and physical health consequences of trauma. Events that are appraised as threatening are more strongly associated with high demands and lead to biological changes like increased peripheral vascular resistance [46] and higher reactive levels of cortisol [47–51].

The trauma experience question in this study was taken from DSM-IV where the stressor is defined as an event that has been experienced or witnessed. In the DSM-5 criteria the definition of the stressor has been broadened to include learning that the traumatic event(s) occurred to a close family member or friend (with the actual or threatened death being either violent or accidental) and experiencing repeated or extreme exposure to aversive details of the traumatic event. Whilst we were unable to assess the DSM-5 criteria in the current study, future research may find that this change in wording influences responses on the trauma screening question.

### Strengths and limitations

This study was done with extensive efforts to access individuals residing in a diverse urban setting with a high level of social deprivation and higher levels of crime. We used a checklist for objective measure of the trauma event exposure and allowed participants to appraise the experience and give subjective judgement. Both these measures are taken at the same point in time together with the mental health assessment. The method used in this study may also overcome potential recall bias in the subjective assessment of events because participants completed a prompted checklist which may facilitate memory recall of events before the subjective screening question was administered. Our results provide insight into the extent of exposure to trauma and factors influencing appraisal of traumatic experience in this community. The interpretation of the results should take into account the limitations of this study. The list of life events is not exhaustive and did not account for the severity of each event or a measure of perceived threat. Further, the recency, frequency and the exact circumstances of each event was not collected. Furthermore, by administering the trauma checklist before the subjective screening question, this could have acted as a memory prompt and consequently influenced responses on the screening question.

## Conclusions

Our study demonstrates that gender, early life trauma and greater accumulation of life events may influence an individual’s judgement concerning the severity of threat posed by an event. This subjective processes contributes to individual differences in the screening of trauma and to discrepancies between participants’ reports of traumatic life events on a checklist, compared to when using a DSM event screening tool. Our findings did not support our hypothesis that current symptoms of CMD would account for differences in reporting of traumatic experiences. However, our results suggest that epidemiological approaches to assess PTSD which require participants to make subjective judgements in defining trauma, may capture individual differences in this perception process, for example by gender and previous trauma experience.

Subjective interpretation of a trauma is crucial in relation to the subsequent psychobiological response to that trauma. Understanding the mechanisms underlying the connection between the cognitive appraisal process as it relates to the factors identified in this study (gender, multiple trauma and early life trauma), would help to build more effective models of intervention addressing the mental and physical health consequences of trauma.

## References

[1] Burnam MA, Stein JA, Golding JM, Siegel JM, Sorenson SB, Forsythe AB (1988). Sexual assault and mental disorders in a community population. J Consult Clin Psychol.

[2] Byrne CA, Resnick HS, Kilpatrick DG, Best CL, Saunders BE (1999). The socioeconomic impact of interpersonal violence on women. J Consult Clin Psychol.

[3] Felitti VJ, Anda RF, Nordenberg D, Williamson DF, Spitz AM, Edwards V (1998). Relationship of childhood abuse and household dysfunction to many of the leading causes of death in adults. The Adverse Childhood Experiences (ACE) Study. Am J Prev Med.

[4] Breslau N, Davis GC, Andreski P, Peterson E (1991). Traumatic events and posttraumatic stress disorder in an urban population of young adults. Arch Gen Psychiatry.

[5] Davidson JRT, Hughes D, Blazer DG, George LK (1991). Post-traumatic stress disorder in the community: an epidemiological study. Psychol Med.

[6] Helzer JE, Robins LN, McEvoy L (1987). Post-traumatic stress disorder in the general population. N EnglJ Med.

[7] Hepp U, Gamma A, Milos G, Eich D, Ajdacic–Gross V, Rössler W (2006). Prevalence of exposure to potentially traumatic events and PTSD. Eur Arch Psychiatry Clin Neurosci.

[8] Kessler R, Sonnega A, Bromet E, Hughes M, Nelson C (1995). Posttraumatic stress disorder in the National Comorbidity Survey. Arch Gen Psychiatry.

[9] Perkonigg A, Kessler RC, Storz S, Wittchen HU (2000). Traumatic events and post-traumatic stress disorder in the community: prevalence, risk factors and comorbidity. Acta Psychiatr Scand.

[10] Dorrington S, Zavos H, Ball H, McGuffin P, Rijsdijk F, Siribaddana S (2014). Trauma, post-traumatic stress disorder and psychiatric disorders in a middle-income setting: prevalence and comorbidity. Br J Psychiatry.

[11] Frissa S, Hatch S, Gazard B, Fear N, Hotopf M (2013). Trauma and current symptoms of PTSD in a South East London community. Soc Psychiatry Psychiatr Epidemiol.

[12] American Psychiatric Association (2013). Diagnostic and statistical manual of mental disorders.

[13] Blaney PH (1986). Affect and memory: a review. Psychol Bull.

[14] Bower GH (1981). Mood and memory. Am Psychol.

[15] McNally RJ (2003). Psychological mechanisms in acute response to trauma. Biol Psychiatry.

[16] Breslau N, Kessler RC, Chilcoat HD, Schultz LR, Davis GC, Andreski P (1998). Trauma and posttraumatic stress disorder in the community: the 1996 Detroit area survey of trauma. Arch Gen Psychiatry.

[17] McNaughton N, Corr PJ (2004). A two-dimensional neuropsychology of defense: fear/anxiety and defensive distance. Neurosci Biobehav Rev.

[18] Ehlers A, Clark DM (2000). A cognitive model of posttraumatic stress disorder. Behav Res Ther.

[19] Cromer L, Smyth J (2010). Making meaning of trauma: trauma exposure doesn’t tell the whole story. J Consult Psychol.

[20] Roemer L, Litz BT, Orsillo SM, Ehlich PJ, Friedman MJ (1998). Increases in retrospective accounts of war-zone exposure over time: the role of PTSD symptom severity. J Trauma Stress.

[21] Schwarz E, Kowalski J, McNally R (1993). Malignant memories: Post-traumatic changes in memory in adults after a school shooting. J Trauma Stress.

[22] Southwick SM, Morgan CA, Nicolaou AL, Charney DS (1997). Consistency of memory for combat-related traumatic events in veterans of Operation Desert Storm. Am J Psychiatry.

[23] Wessely S, Unwin C, Hotopf M, Hull L, Ismail K, Nicolaou V (2003). Stability of recall of military hazards over time. Evidence from the Persian Gulf War of 1991. Br J Psychiatry.

[24] Goodman GS, Ghetti S, Quas JA, Edelstein RS, Alexander KW, Redlich AD (2003). A prospective study of memory for child sexual abuse: new findings relevant to the repressed-memory controversy. Psychol Sci.

[25] Widom CS, Morris S (1997). Accuracy of adult recollections of childhood victimization: Part 2. Childhood sexual abuse. Psychol Assess.

[26] Williams LM (1994). Recall of childhood trauma: a prospective study of women’s memories of child sexual abuse. J Consult Clin Psychol.

[27] Sarason IG, Johnson JH, Siegel JM (1978). Assessing the impact of life changes: development of the life experiences survey. J Consult Clin Psychol.

[28] Hatch SL, Frissa S, Verdecchia M, Stewart R, Fear NT, Reichenberg A (2011). Identifying socio-demographic and socioeconomic determinants of health inequalities in a diverse London community: the South East London Community Health (SELCoH) study. BMC Public Health.

[29] Meyer IH (2003). Prejudice as stress: conceptual and measurement problems. Am J Public Health.

[30] Turner RJ, Lloyd DA (1995). Lifetime traumas and mental health: the significance of cumulative adversity. J Health Soc Behav.

[31] Prins A, Ouimette P, Kimerling R, Cameron RP, Hugelshofer DS, Shaw-Hegwer J (2003). The primary care PTSD screen (PC-PTSD): development and operating characteristics. Prim Care Psychiatry.

[32] American Psychiatric Association (1994). Diagnostic and Statistical Manual of Mental Disorders(DSMIV).

[33] Lewis G, Pelosi AJ, Araya R, Dunn G (1992). Measuring psychiatric disorder in the community: a standardized assessment for use by lay interviewers. Psychol Med.

[34] LP S, StataCorp (2009). Stata statistical software. Release 11.

[35] Bremner J, Southwick S, Johnson D, Yehuda R, Charney D (1993). Childhood physical abuse and combat-related posttraumatic stress disorder in Vietnam veterans. Am J Psychiatry.

[36] Breslau N, Chilcoat HD, Kessler RC, Davis GC (1999). Previous exposure to trauma and PTSD effects of subsequent trauma: results from the Detroit Area Survey of Trauma. Am J Psychiatry.

[37] Coid J, Petruckevitch A, Feder G, Chung W-S, Richardson J, Moorey S (2001). Relation between childhood sexual and physical abuse and risk of revictimisation in women: a cross-sectional survey. Lancet.

[38] Iversen AC, Fear NT, Ehlers A, Hacker Hughes J, Hull L, Earnshaw M (2008). Risk factors for post-traumatic stress disorder among UK Armed Forces personnel. Psychol Med.

[39] Burstein A (1985). Posttraumatic stress disorder. J Clin Psychiatry.

[40] Engelhard IM, van den Hout MA, Arntz A (2001). Posttraumatic stress disorder after pregnancy loss. Gen Hosp Psychiatry.

[41] Czarnocka J, Slade P (2000). Prevalence and predictors of post-traumatic stress symptoms following childbirth. Br J Clin Psychol.

[42] Nice DS, Garland CF, Hilton SM, Baggett JC, Mitchell RE (1996). Long-term health outcomes and medical effects of torture among US Navy prisoners of war in Vietnam. JAMA.

[43] Basoglu MPM, Paker O, Ozmen E, Marks I, Incesu C, Sahin D (1994). Psychological effects of torture: a comparison of tortured with nontortured political activists in Turkey. Am J Psychiatry.

[44] Perry SDJ, Musngi G, Frances A, Jacobsberg A (1992). Predictors of posttraumatic stress disorder after burn injury. Am J Psychiatry.

[45] Schnyder UMH, Klaghofer R, Claus Buddeberg C (2001). Incidence and prediction of posttraumatic stress disorder symptoms in severely injured accident victims. Am J Psychiatry.

[46] Tomaka J, Blascovich J, Kelsey RM, Leitten CL (1993). Subjective, physiological, and behavioral effects of threat and challenge appraisal. J Pers Soc Psychol.

[47] Denson TF, Spanovic M, Miller N (2009). Cognitive Appraisals and Emotions Predict Cortisol and Immune Responses: A Meta-Analysis of Acute Laboratory Social Stressors and Emotion Inductions. Psychol Bull.

[48] Harvey A, Nathens AB, Bandiera G, LeBlanc VR (2010). Threat and challenge: cognitive appraisal and stress responses in simulated trauma resuscitations. Med Educ.

[49] Lundberg U, Frankenhaeuser M (1980). Pituitary-adrenal and sympathetic-adrenal correlates of distress and effort. J Psychosom Res.

[50] Olff M, Langeland W, Gersons BPR (2005). Effects of appraisal and coping on the neuroendocrine response to extreme stress. Neurosci Biobehav Rev.

[51] Vaernes R, Ursin H, Darragh A, Lambe R (1982). Endocrine response patterns and psychological correlates. J Psychosom Res.

